# Holographic Characteristics of Photopolymers Containing Different Mixtures of Nematic Liquid Crystals

**DOI:** 10.3390/polym11020325

**Published:** 2019-02-13

**Authors:** Sandra Fenoll, Francisco Brocal, José David Segura, Manuel Ortuño, Augusto Beléndez, Inmaculada Pascual

**Affiliations:** 1 Instituto Universitario de Física Aplicada a las Ciencias y las Tecnologías, Universidad de Alicante, Apartado 99, E03080 Alicante, Spain; fenoll.sandra@gmail.com (S.F.); francisco.brocal@ua.es (F.B.); jdavidsegura90@gmail.com (J.D.S.); a.belendez@gcloud.ua.es (A.B.); pascual@ua.es (I.P.); 2 Departamento de Física, Ingeniería de Sistemas y Teoría de la Señal, Universidad de Alicante, Apartado 99, E03080 Alicante, Spain; 3 Departamento de Óptica, Farmacología y Anatomía, Universidad de Alicante, Apartado 99, E03080 Alicante, Spain

**Keywords:** holography, photopolymer, holographic recording materials, holographic polymer dispersed liquid crystal

## Abstract

A holographic polymer dispersed liquid crystal (HPDLC) is used to record holographic diffraction gratings. Several mixtures of nematic liquid crystals (LC) are used as components of the HPDLC to evaluate their influence in static and dynamic basic properties. The diffraction efficiency obtained in the reconstruction of the holograms is evaluated to compare the influence of the different LC. Additionally, the samples are exposed to a variable electric field and the diffracted light intensity as a function of the applied voltage is measured to evaluate the influence of the LC. The results obtained show significant differences depending on the LC incorporated to the photopolymer.

## 1. Introduction

Photopolymers are a type of holographic recording material that are used in many optical applications due to their flexibility, low cost, and easy preparation [1,2,3,4,5,6]. In these materials, different physical properties must usually be optimized, for example, energetic sensitivity to decrease the energy required during the recording process, spectral sensitivity to select a specific wavelength, or the spatial frequency to obtain high fidelity in the reconstruction of the hologram [7,8].

Photopolymers have a photoinitiator that absorbs light from the recording laser and generates radicals that start the free-radical polymerization reaction of monomers. For a holographic recording, the mechanism of hologram growing includes modulation of the refractive index between polymerized and non-polymerized areas, corresponding to the “exposed” and “unexposed” areas, respectively, in the diffraction grating generated by the interference of the recording laser beams [7,9].

The combination of liquid crystals with photopolymers adds a new characteristic, the capacity to change the electro-optical properties with an electric field. The liquid crystal molecules add optical anisotropy to the composite material and its response can be modified by the electric field applied [10,11,12,13,14,15]. Holographic polymer dispersed liquid crystals are known as HPDLC. When these composites are exposed to a holographic recording they undergo a photopolymerization-induced phase separation process or PIPS in which the liquid crystal molecules diffuse to unexposed areas of the diffraction grating where they can be oriented with an electric field [16,17,18,19,20]. Depending on the application, different physical parameters must be evaluated [21].

In this work, several mixtures of nematic liquid crystals (LC) are used as components of a HPDLC to evaluate their influence in static and dynamic basic properties. The LC used are commercial products not developed specifically for their application in HPDLC and some of them have been discontinued by the manufacturers. The main physical properties of each LC mixture are specified in Table 1.

We have prepared an electro-optical device with each solution of HPDLC containing a different LC. After, a diffraction grating is recorded by means of laser exposure and different parameters are evaluated in order to identify differences between the LC.

The diffraction efficiency obtained in the reconstruction of the holograms is evaluated to compare the influence of the different LC. Additionally, the samples are exposed to an electric field and the diffracted light intensity as a function of the applied voltage is measured to evaluate the differences due to the different LC. Finally, the devices are exposed to incoherent white light in order to stabilize the grating and to complete the polymerization process and the previously named parameters are measured again. The results obtained show significant differences depending on the LC incorporated into the photopolymer. These differences are important taking account potential practical applications for these photopolymers and HPDLC devices.

## 2. Experimental Section

### 2.1. Preparation of the Composite Solutions and Devices

We use a type of HPDLC for which formulation is very flexible and it has been used in many works. Monomers, initiators, and additives can be changed to obtain photopolymers with different responses to wavelength, light intensity, polymerization, etc. The bibliography is very extensive, some representative works can be consulted in the references [13,22,23,24,25]. We have slightly modified the basic components of this composite in previous works [26,27].

Several formulations of HPDLC have been prepared with a different LC mixture to study the electro-optical properties of the devices. The LC used are BL087, K15, MLC6882, TL213, and BL038 from Merck, QYPDLC-036 (LC036) from Qingdao QY Liquid Crystal Co., Ltd. (Qingdao, China). They are mixtures of 4-cyanobiphenyls with alkyl chains of different lengths and different substituents. They are designed for electro-optic displays but not specifically for HPDLC. PBC is 4′-Pentyl-4-biphenylcarbonitrile (5CB) with a purity of 98% from Sigma-Aldrich (St. Louis, MO, USA). Table 1 shows the main physical properties of the LC mixtures. 

Where

- Δn is the birefringence, which is defined as the difference between the extraordinary refractive index (light polarized parallel to the director vector) and the ordinary refractive index (light polarized perpendicular to the director vector). It has an influence on the diffraction efficiency obtained by the HPDLC because molecules of LC are diffused to unexposed areas during PIPS. Without an electric field, these areas have an average value of refractive index between the extraordinary index and the ordinary index.
- n_0_ is the ordinary refractive index. This parameter is of interest when compared to the refractive index of the polymer when an electric field is applied to the HPDLC device.
- Δε is the dielectric anisotropy defined as the difference of the dielectric constants parallel and perpendicular to the director vector of the nematic phase. It has an influence on the holographic properties of HPDLC device under the action of an electric field.

The values have been obtained from the documentation published by the manufacturers and bibliography [28,29,30,31,32].

For PBC, the manufacturer does not offer the concrete values of Δn and Δε. These values must be similar to those of K15 since the chemical composition is the same although the degree of purity could be different.

The monomer used was dipentaerythritol penta/hexa acrylate (DPHPA) with a refractive index n = 1.490. *N*-methyl-2-pyrrolidone (NMP) was used as a solvent, octanoic acid (OA) as cosolvent and surfactant, ethyl eosin (YEt) as a dye, and *N*-phenyl glycine (NPG) as an initiator. All components were obtained from Sigma-Aldrich (St. Louis, MO, USA). Table 2 shows the composition of the composite.

LC is each one of the LC mixtures. The solution was made by mixing the substances under red light where the material is not sensitive. An ultrasonic bath with a temperature of 35 °C was used to homogenize the solution. The solution was deposited between two indium tin oxide conductive glasses with 1 mm thick and separated with 13 µm glass hollow microspheres from Sigma-Aldrich (St. Louis, MO, USA). 

### 2.2. Holographic Set Up

**Figure 1:**
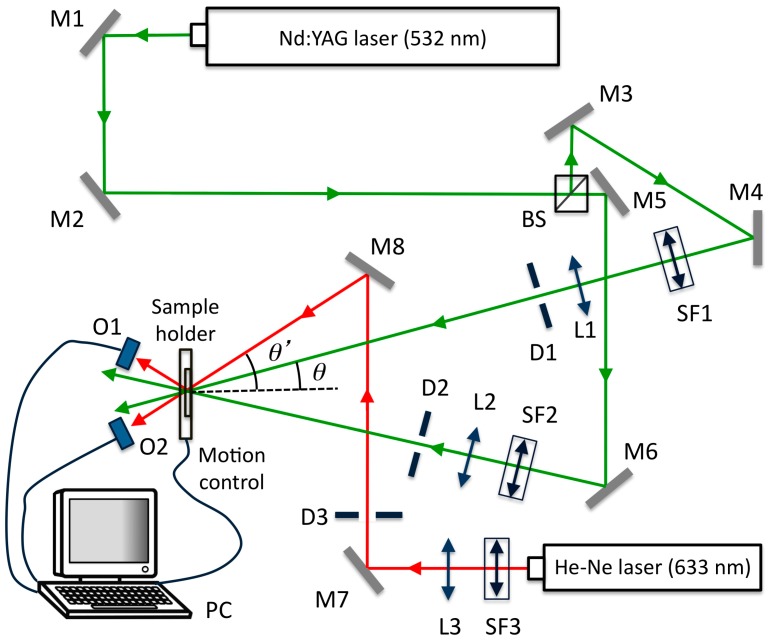
Holographic set-up. BS: Beamsplitter, M_i_: mirror, SF_i_: spatial filter, L_i_: lens, D_i_: diaphragm, Oi: optical power meter, PC: data acquisition.

We use the holographic set up shown in Figure 1. The devices prepared were exposed to a diode-pumped solid-state laser from MKS Instruments, Inc. (Spectra-Physics Excelsior One 532 multi mode, MA, USA) with a wavelength of 532 nm in order to record an unslanted diffraction grating in the HPDLC device. The laser beam with TE polarization was separated into two beams with a light intensity ratio of 1:1. We used lenses and spatial filters to obtain two beams with a diameter of 1 cm. The object and reference beams were recombined on the sample at an angle θ = 16.0° to the normal using mirrors. The spatial frequency was 1036 lines/mm. The working light intensity was 2.2 mW/cm^2^. The diffracted and transmitted intensity were monitored in real time with a 632.8 nm He-Ne laser positioned at Bragg’s angle θ‘ = 19.1° where the material is not sensitive [33]. During the recording, the temperature was 22 °C and the recording time was adjusted to 40 s after an optimization process to obtain the maximum diffraction efficiency without overmodulation.

**Figure 2:**
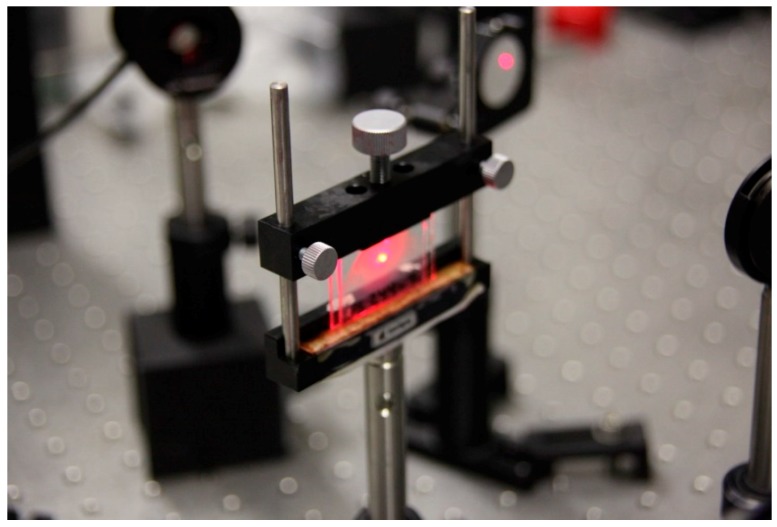
Reconstruction laser beam passing through the sample.

Figure 2 shows the reconstruction beam from the He-Ne laser passing through the sample.

After holographic recording, we used a Tektronix AFG3022B dual channel arbitrary function generator (Tektronix Inc., Beaverton, OR, USA) and a N4L voltage amplifier (Newtons4th Ltd, Leicester, UK) to expose the devices to 1 kHz square wave signal voltage. Root mean square (RMS) voltage and RMS electric current intensity are measured with specific multimeters for the frequency range were used. The diffracted light intensity as a function of the RMS voltage was monitored with the He-Ne laser.

In this type of composite, a photopolymerization reaction takes place in the exposed areas of the diffraction grating and a highly reticulated polymer network is generated. During the PIPS, the liquid crystal diffuses to the unexposed areas where it remains as droplets [19,34].

## 3. Results and Discussion

### 3.1. Variation of the Electric Current Intensity with the Voltage

After recording the diffraction grating, each HPDLC device is exposed to the electric field. The voltage increase is manually done. The samples remain in each voltage for the time necessary until the value of the electric current intensity (I) stabilizes (typically 1 s).

**Figure 3:**
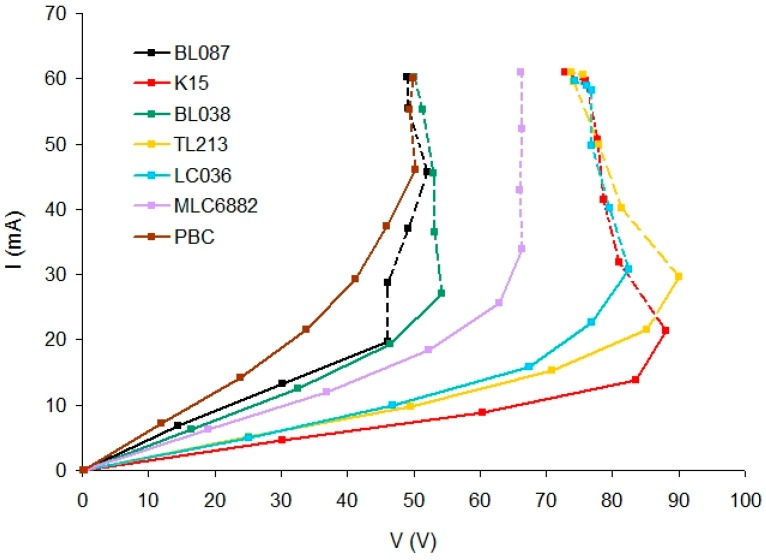
Electric current intensity versus root mean square (RMS) voltage for holographic polymer dispersed liquid crystal (HPDLC) devices with different LC.

Figure 3 shows the variation of the electric current intensity that passes through the device versus RMS voltage.

It can be seen that the electric current intensity progressively increases with the applied voltage up to a threshold voltage value. From this value (dashed line), I increases drastically. Three substances contained in the polymer (Table 2) can produce ions in an aqueous medium: YEt, NPG, and OA. These molecules are not dissociated in a non-aqueous medium as in this polymer because the possible ions generated (K^+^ for YEt and H^+^ for OA and NPG) are not stable in this medium. This polymer does not have any component that can act as a supporting electrolyte and, therefore, with a low voltage, the conductivity is low and the polymer is a dielectric.

When a sufficiently high voltage is applied, the conductivity increases because these substances start to dissociate and they could have significant importance in increasing the electric current. Moreover, impurities contained in these substances and also in LC, NMP, and monomer could play a role in supporting the electric current. For example, PBC and k15 with the same nematic component 5CB produce a different result. Although in this case, it is not possible to rule out potential differences during the preparation of the samples because the angular response for PBC could not be obtained (Figure 7).

There are deviations in the points of the dashed lines. Points whose measured voltage is lower than the voltage of preceding points although during the experiment the amplitude of the voltage signal is progressively increased from the signal generator.

This could be related to changes in the permittivity of the LC due to the electric current. A sufficiently high electric current intensity could influence the orientation of the LC molecules modifying their permittivity, which influences the electric current.

Table 3 shows the threshold voltage for each type of LC.

**Figure 4:**
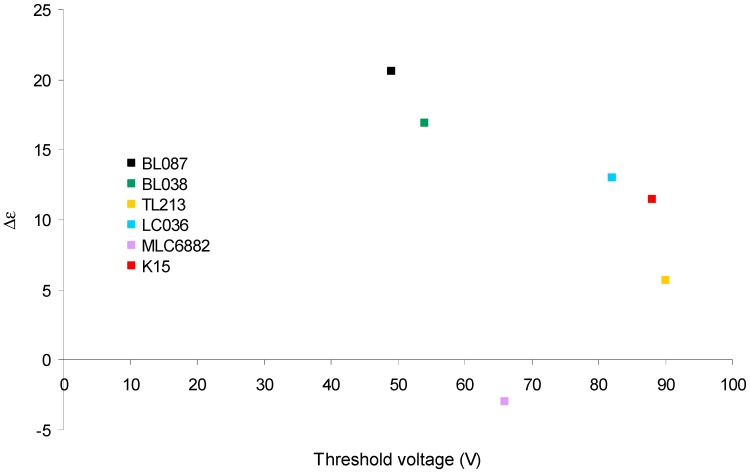
Δε versus the threshold voltage.

Figure 4 shows the Δε versus the threshold voltage. It is observed that for LC with Δε > 0 (Table 1), this value decreases when the threshold voltage is increased. MLC6882 has a different behavior due to Δε < 0.

Since Δε is related to the response of the LC molecules under the action of an electric field, the magnitude of Δε affects the electrical behavior of the device. The device is more sensitive to the electric field if the LC used has a high Δε value.

### 3.2. Variation of the Diffracted Light Intensity with the Voltage

After recording the diffraction grating, each HPDLC device is exposed to the electric field and simultaneously the hologram is reconstructed by means of the He-Ne laser. In this experiment, the voltage is progressively increased whereas the diffracted light intensity of the order +1 is measured with a radiometer. The voltage increase is manually done. The samples remain in each voltage for the time necessary until the value of the diffracted light intensity (Id) stabilizes (typically 1 s).

**Figure 5:**
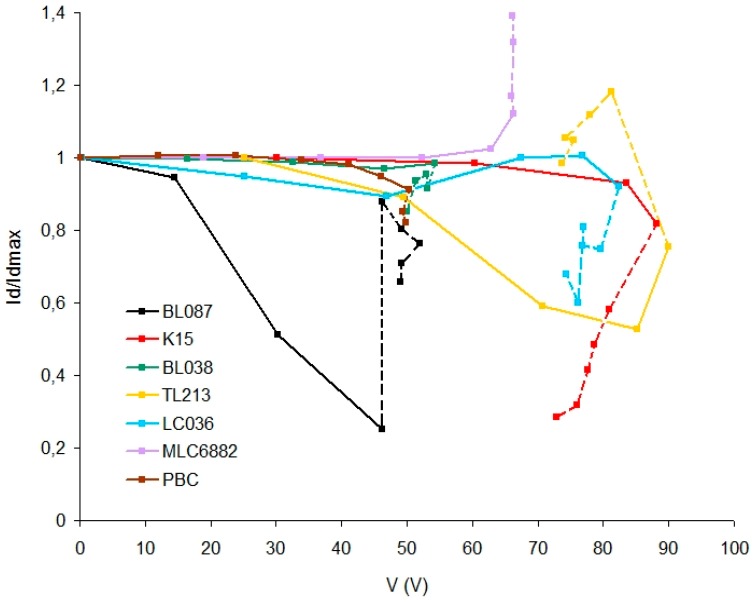
Diffracted light intensity divided by the maximum diffracted light intensity versus RMS voltage for each HPDLC device containing a different LC.

Figure 5 shows Id divided by the maximum diffracted light intensity (Idmax) versus RMS voltage for each HPDLC device containing a different LC. For each graph, the line is dashed after the threshold voltage.

It is observed that all the samples respond to a threshold voltage lower than 100 V, which is a relatively small value for a HPDLC device [35]. This is because the unexposed areas of the diffraction grating remain without polymerizing after holographic recording and the droplets of liquid crystal are in a liquid phase with little restriction of movement, depending only on the viscosity of the photopolymer. This suggests that the droplets can move or rotate, making it difficult for the director vector of the LC molecules to reach a defined average position depending on the applied voltage. As a consequence, only with values close to the threshold voltage will the Id/Idmax ratio decrease. There are two exceptions, TL213 and BL087 in which the Id/Idmax ratio decreases significantly (0.55 and 0.25 respectively) before the threshold voltage is reached. This result is very interesting due to the adverse condition in which the devices are working with the unexposed areas without polymerizing, achieving a great decrease in the Id/Idmax ratio for a very small voltage: 47 V for BL087 and 88 V for TL213.

After the threshold voltage (dashed lines), there are deviations produced by the relatively high electric current intensity. As can be seen in Figure 3, a sufficiently high electric current intensity could influence the orientation of the LC molecules modifying their permittivity which influences the electric current, voltage, and diffracted light intensity.

Subsequently, the samples were exposed to incoherent white light by means of a halogen bulb (1100 lx, 15 min) and the intensity of the diffracted light versus the voltage was measured again.

**Figure 6:**
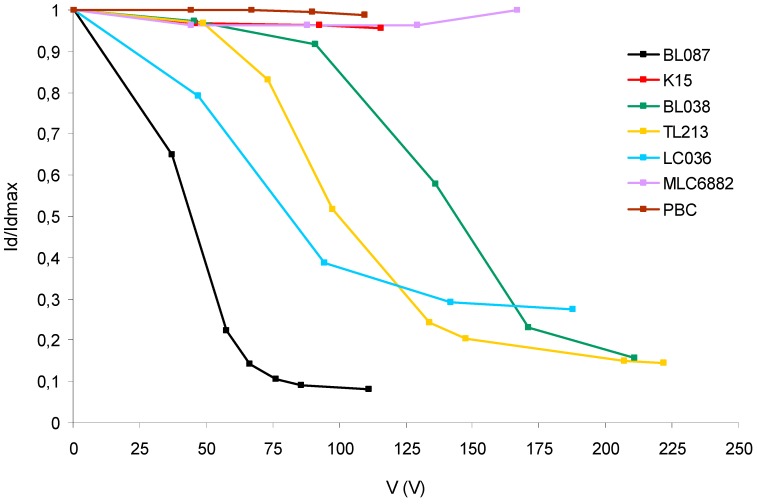
Diffracted light intensity divided by the maximum diffracted light intensity versus RMS voltage for each HPDLC device after the bleaching process.

Figure 6 shows the intensity of diffracted light of the order +1 divided by the maximum diffracted light intensity (Id/Idmax) versus the RMS voltage for each sample after the bleaching process.

As can be seen in Figure 6, all the graphs now have the typical behavior of a HPDLC device that is quite different from those in Figure 5. The differences between Figure 5 and Figure 6 can be explained because the samples are subjected to the bleaching process. The unexposed areas of the diffraction grating are now polymerized and the polymeric network envelopes the droplets of the liquid crystal hindering their movements. Depending on the LC mixture, significant differences are observed due to the combination of parameters from each LC: Δε, Δn, n_0_.

K15 and PBC are liquid crystals with only one nematic molecule unlike the rest of the LC studied. The result shows a decrease of less than 4% for Id/Idmax with a voltage around 100 V in both LC. It is a much lower decrease than those obtained for the rest of the LC with Δε > 0.

A high value of Δε is needed to obtain a quick orientation of the LC with the electric field. But is not the only necessary condition in HPDLC. The electric field-induced torque is not the only force on the molecules in nematics. The elastic forces, boundary condition, and surface forces between substrates must also be considered related to the orientation of the director vector [36]. The situation is more complex in HPDLC where the LC is dispersed in a polymeric matrix, with a spatial modulation of LC concentration. The shape and size of the LC droplets, their composition and the interaction forces of the droplets with the polymer matrix influence in the orientation of the molecules. Additionally, environmental factors such as temperature play a decisive role in the orientation of LC molecules. Mixtures of liquid crystals are designed by combining nematic molecules with different chain lengths. This makes them have a wide range of working temperatures in which they maintain anisotropic characteristics [37]. K15 and PBC with a single nematic molecule 5CB are very sensitive to the room temperature. The temperature from nematic to crystalline varies according to the source consulted and may be affected by the degree of purity. In general, below 22 °C they undergo a crystalline phase in which the molecules cannot be oriented by the electric field [38,39,40]. A small decrease in ambient temperature may hinder the orientation of the LC molecules with the electric field, taking into account that they are dispersed in droplets inside the polymeric network.

MLC6882 increases the Id/Idmax slightly with the voltage. This is related to the negative value of Δε. A positive dielectric anisotropy will tend to align the molecules parallel to the electric field whereas a negative dielectric anisotropy will tend to align the molecules perpendicular to the electric field and therefore the orientation of the droplets with the electric field decreases the refractive index of the exposed areas of the diffraction grating instead of increasing it. The magnitude of the dielectric anisotropy is related to the strength of the electric-induced torque force in the LC molecule. In addition, the value of Δn is very small and therefore the increase in Id/Idmax with the voltage is small.

BL087 achieves an Id/Idmax < 0.1 with a voltage lower than 100 V. This is the best behavior obtained in this study and it is related to the balance between the parameters of this LC: high Δε, relatively high Δn, and relatively low n_0_ close to the refractive index of the polymer.

The other LC have the expected behavior according to their characteristics but they need a voltage higher than BL087. There are also differences in the slope of the curves, especially with small voltages. This is the speed at which the intensity of diffracted light decreases as a function of the applied voltage and it is influenced by the combination of Δε and Δn. It is necessary to take into account that other factors also influence the response of the devices: temperature variations, differences between devices in the internal structure of droplets created by the PIPS effect, and the difficult preparation of this type of samples subjected to experimental variability to be made by hand.

### 3.3. Reconstruction of the Holograms

Additionally, we have studied the influence of the LC in the recording of the diffraction grating. The volume holograms were reconstructed with the He-Ne laser and the angular response of the order +1 was obtained using a rotating stage. Afterward, the samples were exposed to incoherent white light by means of a halogen bulb (1100 lx, 15 min) in order to stabilize the hologram and they were reconstructed again.

**Figure 7:**
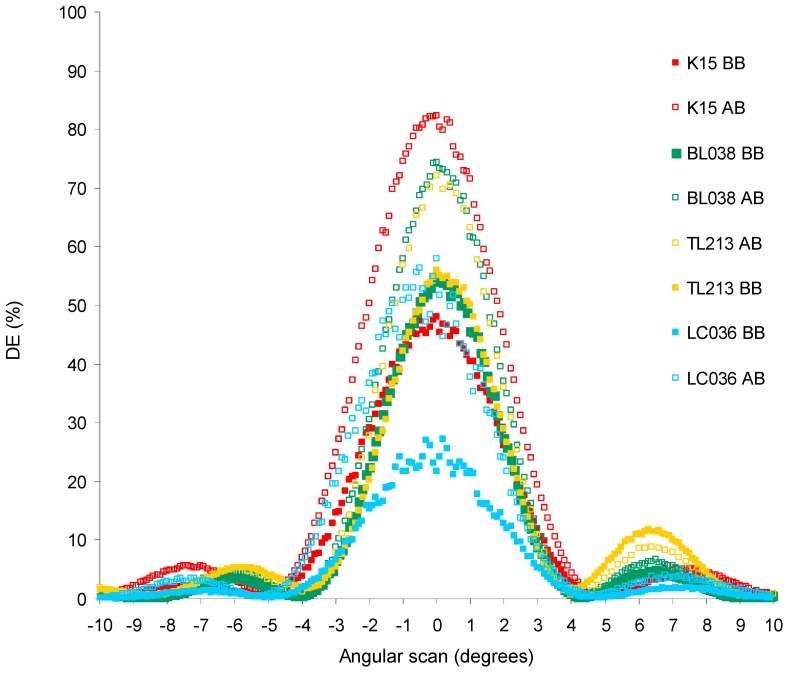
Reconstruction of the hologram before the bleaching process (graphs BB) and after the bleaching process (graphs AB).

Figure 7 shows the reconstruction of the holograms after recording and before the bleaching process (graphs BB) and after the bleaching process (graphs AB).

The diffraction efficiency (DE) is the diffracted light intensity divided by the incident light intensity during the reconstruction of the hologram. DE is obtained for an angular interval of 20° in which the zero value corresponds to the Bragg’s angle.

The graphs for liquid crystals BL087, MLC6882, and PBC have not been obtained. For the other LC, it is observed that the graphs after the bleaching process have a higher maximum diffraction efficiency. This is because the high-intensity white light fully consumes residual monomer in the exposed areas, favoring the molecular diffusion of LC to the unexposed areas. Additionally, the unexposed areas are now polymerized and therefore their refractive index increases.

**Figure 8:**
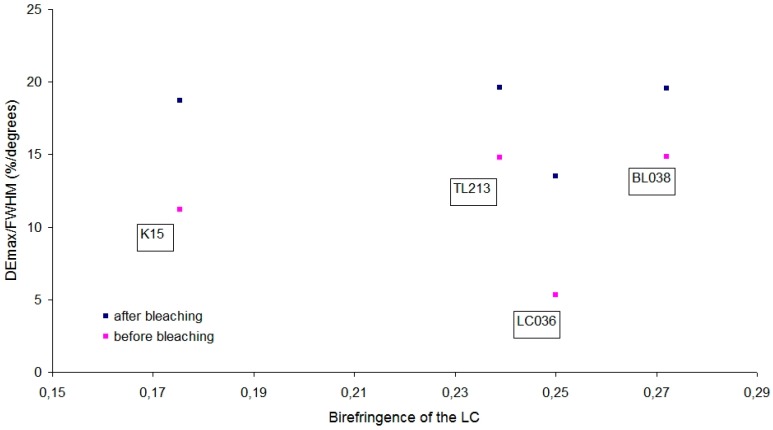
DEmax divided by the FWHM versus the birefringence of the corresponding LC of each graph in Figure 7.

To understand the results from Figure 7, avoiding differences in the width of the curves, the full width at half maximum (FWHM) of each graph has been calculated. Figure 8 shows the maximum diffraction efficiency (DEmax) divided by the FWHM versus the birefringence of the corresponding LC (Table 1) of each graph in Figure 7.

After bleaching, the diffraction grating is stabilized and the results show that the differences are small. DEmax/FWHM is around 19 for all the samples, even for K15 with Δn = 0.1754 lower than the rest [0.2388–0.2720]. TL213 with Δn = 0.2388 obtains a result almost identical to BL038 with Δn = 0.2720.

The sample with LC036 obtains a DEmax/FWHM lower than expected and this points to an experimental problem. The value obtained before bleaching is very small DEmax/FWHM = 5.3 (%/degrees). When the sample is bleached, the value increases significantly up to 13.5, but it is still lower than expected. Since before bleaching DEmax/FWHM was already very small, it is an indication that there was an experimental problem during the sample preparation. The temperature of the solution was probably not optimal during the preparation of the sample or during the recording of the diffraction grating.

For a given holographic application of static type, a greater value of the DEmax can be important. For other applications, a lower value of DEmax but a smaller width of the angular response graph may be more suitable, since that implies a greater optical thickness of the diffraction grating according to the Kogelnik equation for volume holograms and therefore the same DEmax/FWHM [33].

## 4. Conclusions

The influence of different LC in a HPDLC composite has been studied by means of the recording of a diffraction grating and the subsequent analysis of the electro-optical characteristics of the HPDLC device.

After hologram recording, differences in the threshold voltage of the devices depending on the LC have been observed (Figure 3). These differences cover a range of 50 V and it has been related to the different dielectric anisotropy of the LC (Figure 4).

The holographic response of the devices has been studied. Specifically the variation of the intensity of diffracted light as a function of the applied voltage, before and after a white light bleaching process. Before this bleaching process (Figure 5), the intensity of diffracted light varies progressively with the applied voltage for liquid crystals BL087 and TL213, unlike the rest. This result is interesting because the variation is significant for a relatively small voltage value. Nevertheless, if the device is maintained without performing the bleaching process the system would be unstable over time because the LC droplets will tend to collapse.

After the bleaching process (Figure 6), we ascertain experimentally that each device has a different behavior related to the minimum value of Id/Idmax and the shape of the graph in Figure 6 and, more importantly, related to the voltage at which that minimum value is reached. There are significant differences in the values of voltage at the minimum diffracted light. Between the liquid crystal BL087 and TL213, there is a difference of 100 V. This is interesting from the point of view of dynamical practical applications. There are also differences in the speed at which the diffracted light intensity decreases as a function of the applied voltage, especially at small voltages. Therefore, it is confirmed that devices with different LC act in a significantly different way under the action of an electric field. The dielectric anisotropy, birefringence, and the ordinary refractive index are different for each LC and they are the main physical properties implied in the results obtained.

Related to the reconstruction of the holograms without applying an electric field, we found experimentally that each device with different LC obtains a different DEmax (Figure 7). Therefore, there is a direct relationship between the DEmax obtained and the LC used. The DEmax is related to the refractive index modulation according to Kogelnik equation [33]. Each LC has a different Δn value (Table 1) and a LC with high Δn obtains a high refractive index modulation during the holographic recording and therefore a high DEmax according to Kogelnik equation.

DEmax also depends on the optical thickness of the diffraction grating according to Kogelnik equation. We have shown that the combination of DEmax (refractive index modulation) and width of the angular response curve (optical thickness of the diffraction grating) produces a similar result after bleaching for LC with Δε > 0 (Figure 8).

There are many parameters to consider in a HPDLC device and not all have been considered in this work, for example, the response times (t_on_, t_off_) of the devices under exposure to the electric field [41,42]; there are even more to consider if we take into account the different potential applications using a HPDLC as a holographic recording material. However, the results obtained allow a selection of the LC to be used in a HPDLC depending on the character dynamic or static of the application.

## Figures and Tables

**Table 1:** Properties of liquid crystals (LC) mixtures.

	Δn	n_0_	Δε
**BL087**	0.2362	1.5246	20.6
**K15**	0.1754	1.5309	11.5
**MLC6882**	0.0978	--	-3.0
**TL213**	0.2388	1.5271	5.7
**BL038**	0.2720	1.5270	16.9
**LC036**	0.250	1.520	13.0
**PBC**	--	1.532	--

**Table 2:** Composition of the composite material in wt %.

Component	Concentration (wt %)
**DPHPA**	43.00
**LC**	29.50
**YEt**	0.04
**NPG**	0.36
**OA**	8.50
**NMP**	18.60

**Table 3:** Threshold voltage (V) obtained for each HPDLC device with different LC.

BL087	BL038	MLC6882	LC036	K15	TL213	PBC
46	54	66	82	88	90	50
